# Incorporation of Cyano‐Substituted Aromatic Blocks into Naphthalene Diimide‐Based Copolymers: Toward Unipolar n‐Channel Field‐Effect Transistors

**DOI:** 10.1002/smsc.202100016

**Published:** 2021-07-16

**Authors:** Congyuan Wei, Pan Xu, Weifeng Zhang, Yankai Zhou, Xuyang Wei, Yuanhui Zheng, Liping Wang, Gui Yu

**Affiliations:** ^1^ Beijing National Laboratory for Molecular Sciences CAS Research/Education Center for Excellence in Molecular Sciences Institute of Chemistry Chinese Academy of Sciences Beijing 100190 P. R. China; ^2^ School of Chemical Sciences University of Chinese Academy of Sciences Beijing 100049 P. R. China; ^3^ School of Material Science and Engineering University of Science and Technology Beijing Beijing 100083 P. R. China

**Keywords:** cyano-substituted acceptor units, electron mobility, field-effect transistors, naphthalene diimide-based copolymers, n-type polymeric semiconductors

## Abstract

The unipolar n‐type polymeric semiconductors are crucial for the development of complementary inverters and complementary logic circuits. To achieve this target, the polymer skeleton should be electron‐deficient, which guarantees the energy‐level alignment between the lowest unoccupied molecular orbital energy level of polymeric materials and the work function of electrode, further permitting effective electron injection. Different from the introduction of the *sp* ^2^‐hybridized nitrogen atoms and fluorine atoms, cyano‐substituted aromatic blocks are synthesized and further copolymerized with naphthalene diimide (NDI) unit, affording a series of copolymers of **PNDI‐BTCN**, **PNDI‐TVTCN**, and **PNDI‐SVSCN**. The photophysical, electrochemical, and thermal properties of all the copolymers are systematically investigated, and their semiconducting performance is studied by fabricating field‐effect transistors and tested under atmosphere. All the polymers exhibit unipolar n‐type semiconducting performance because of the synergetic effect of strong electron‐withdrawing NDI units and cyano‐substituted aromatic blocks. The highest mobility of 0.20 cm^2^ V^−1^ s^−1^ is obtained. Moreover, theoretical simulation and thin‐film characterization are conducted to reveal the difference in semiconducting performance among the three polymeric materials.

## Graphical abstract

A series of polymers of **PNDI‐BTCN**, **PNDI‐TVTCN**, and **PNDI‐SVSCN** are synthesized and studied systematically. All the materials behave as unipolar n‐type semiconductors because of the synergetic effect of strong electron‐withdrawing naphthalene diimide (NDI) unit and cyano‐substituted aromatic blocks. Owing to the superior crystallinity and surface morphology, devices based on **PNDI‐SVSCN** perform the highest mobility of 0.20 cm^2^ V^−1^ s^−1^.

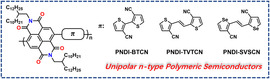

## 1 Introduction

Organic semiconducting materials have gained a lot of attention of industry and academia in recent decades for their potential applications in organic electronics, in which they could be used as the active materials to construct large‐area, flexible, and stretchable devices, via solution‐based processing techniques such as dip‐coating, spin‐coating, inkjet printing, and so on.^[^ ^1^, ^2^, ^3^ ^]^ Organic field‐effect transistor (OFET) is one of the promising organic electronic devices.^[^ ^4^, ^5^, ^6^ ^]^ Specifically, the carrier mobility can be tuned by regulating the gate voltage. OFETs act as the promising devices in flexible displays, radio frequency identify tag, sensors, and so on in the foreseeable future.^[^ ^7^, ^8^, ^9^ ^]^ The rapid development of organic electronics is attributed to the exploration of conjugated polymers and the structure–property relationships, which mainly depends on the incorporation of new monomers into the polymeric skeleton.^[^ ^10^, ^11^, ^12^, ^13^, ^14^ ^]^ By designing the molecular structure reasonably, the carrier transport type could be tuned on purpose and high‐performance semiconductors would be achieved.^[^ ^15^, ^16^, ^17^, ^18^ ^]^

**Figure 1:**
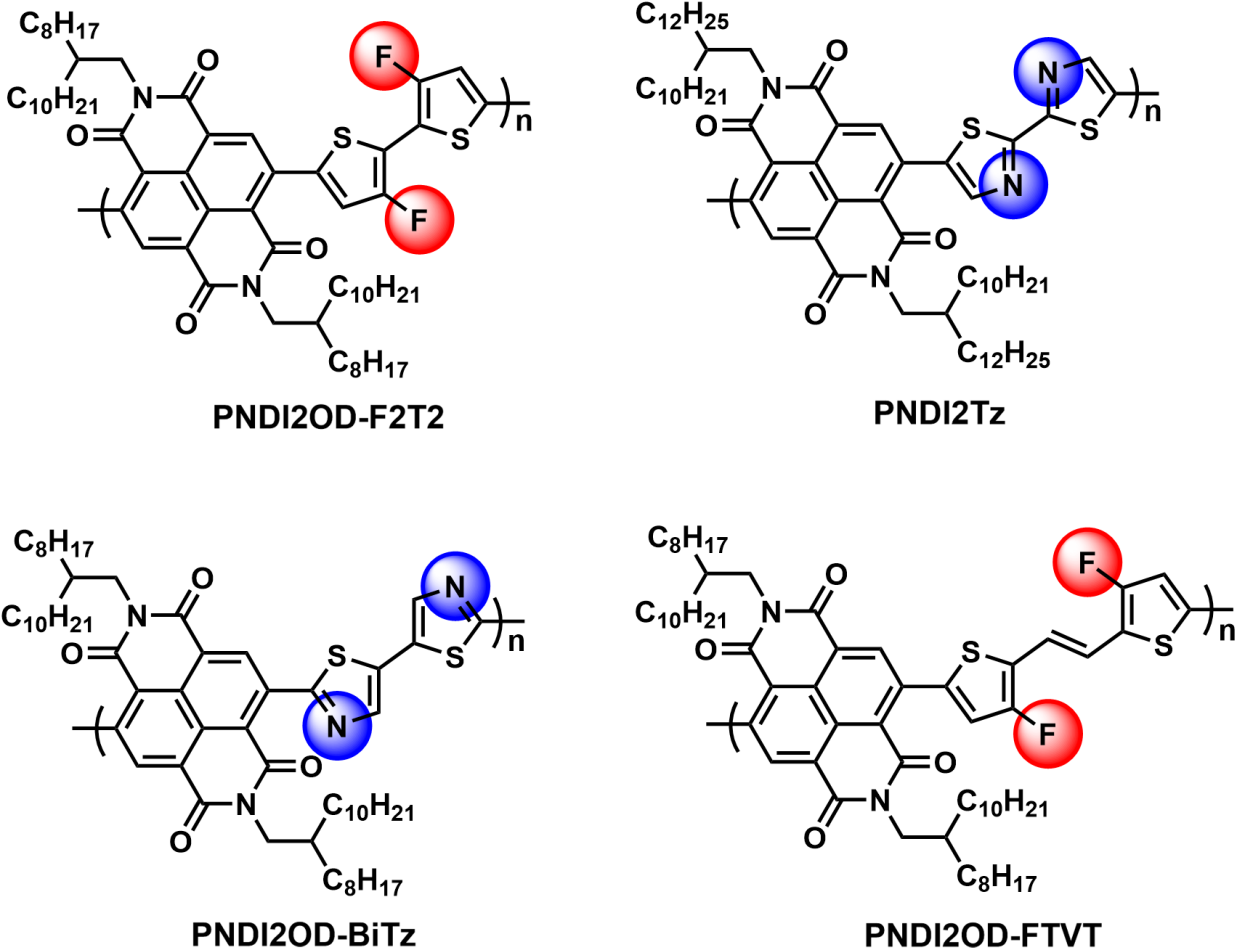
The representative n‐type polymeric semiconducting materials based on NDI units.

Compared with p‐type polymeric semiconductors, the development of n‐type counterparts lags far behind, probably due to the difficulty in synthesizing molecules with low lowest unoccupied molecular orbital (LUMO) energy levels. As far as we know, the most typical monomer is naphthalene diimide (NDI) in constructing n‐type semiconductors for its four strong electron‐withdrawing carbanyl groups and the imide units, which enables the resulting molecules possessing matched energy level and good solubility and further benefits the electron injection and device fabrication.^[^ ^19^, ^20^ ^]^ For example, copolymer of PNDI2OD‐F2T2 reported by Cho et al. showed the electron‐dominated ambipolar performance.^[^ ^21^ ^]^ Substitution of the C–H band in the conjugation backbone with the *sp* ^2^‐hybridized nitrogen atoms would also contribute to the electron‐transporting performance for its strong electron‐withdrawing ability. For instance, Reichmanis et al. and Sommer et al. independently synthesized copolymer of PNDI2Tz by introducing 2,2′‐bithiazole into polymer molecular skeleton.^[^ ^22^, ^23^ ^]^ The resulting polymers possess electron affinity of around 4.10 eV, which is lower than that of the bithiophene analogue P(NDI2OD‐T2).^[^ ^24^ ^]^ And thus the corresponding transistors exhibited electron mobility of up to 0.85 cm^2^ V^−1^ s^−1^. By changing the substitution position of *sp* ^2^‐hybridized nitrogen atoms, the copolymerization between 5,5′‐bithiazole and NDI affords PNDI2OD‐BiTz.^[^ ^25^, ^26^ ^]^ This polymer also showed a LUMO energy level of around −4.0 eV and the fabricated devices displayed unipolar n‐type semiconducting performance. Apart from the modification of the 2,2′‐bithiophene, (*E*)‐1,2‐di(thiophen‐2‐yl)ethene was also decorated with fluorine atoms at the 3,3′‐positions. The obtained copolymer of PNDI2OD‐FTVT has decreased LUMO energy level, and the electron‐dominated ambipolar semiconducting performance was achieved.^[^ ^27^ ^]^ The mentioned strategies of substitution of *sp* ^2^‐hybridized nitrogen atoms or fluorine atoms provide effective approaches for lowing LUMO energy levels (**Figure** **1**). However, these strategies are insufficient in the development of unipolar n‐type polymeric molecules.

Similar to the electron‐withdrawing units of *sp* ^2^‐hybridized nitrogen atoms and fluorine atoms, the introduction of cyano groups is also a feasible method in modulation of majority of carrier transport type as demonstrated by Hwang et al.^[^ ^28^ ^]^ Recently, our group reported a series of unipolar n‐type conjugated polymers based on the cyano‐substituted (*E*)‐1,2‐di(thiophen‐2‐yl)ethene, i.e., (*E*)‐1,2‐bis(3‐cyanothiophene‐2‐yl)ethene.^[^ ^29^ ^]^ Guo et al. also synthesized n‐type polymeric semiconductors by adopting cyano‐functionalized strategy.^[^ ^30^ ^]^ These results demonstrate that n‐type semiconductors would be achieved via this method. To further explore unipolar n‐type polymeric semiconductors and provide guidance for the development of this field, we synthesized a series of cyano‐substituted monomers of 3,3′‐dicyano‐2,2′‐bithiophene (BTCN), (*E*)‐1,2‐bis(3‐cyanothiophene‐2‐yl)ethene (TVTCN), and (*E*)‐1,2‐bis(3‐cyanoselenophene‐2‐yl)ethene (SVSCN). These monomers were functionated and then copolymerized with NDI units, providing **PNDI‐BTCN**, **PNDI‐TVTCN**, and **PNDI‐SVSCN**. The photophysical, electrochemical, and thermal properties were systematically investigated. The semiconducting performances of all the polymers were evaluated by fabricating field‐effect transistors (FETs). All the synthesized polymers display unipolar n‐type semiconducting performance, and devices based on **PNDI‐SVSCN** showed the highest mobility of 0.20 cm^2^ V^−1^ s^−1^. In addition, the molecular geometries were analyzed by performing theoretical simulations.

## 2 Results and Discussion

### 2.1 Synthesis and Thermal Properties

**Scheme 1:**
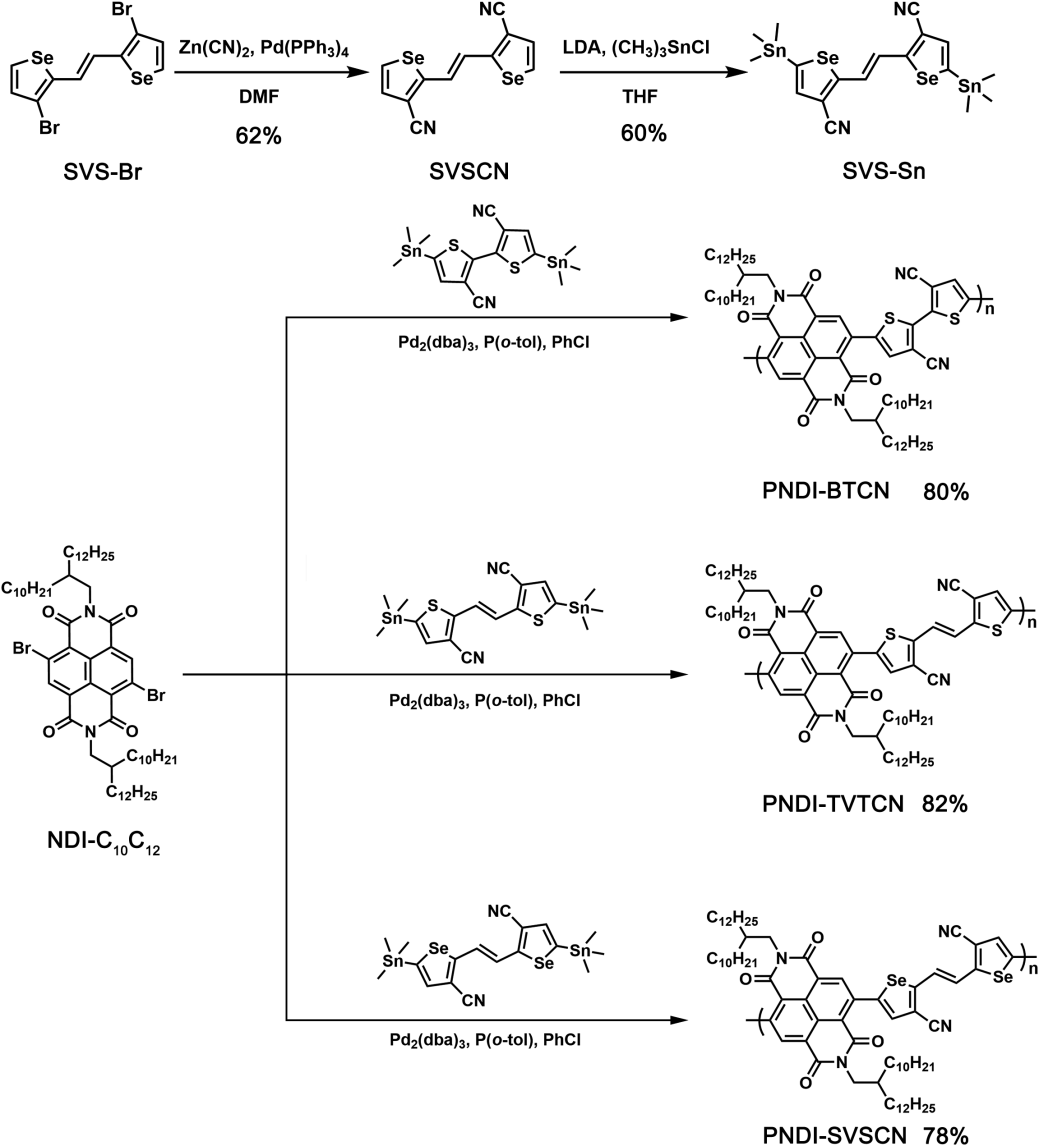
Molecular structures and the synthetic routes of **PNDI‐BTCN**, **PNDI‐TVTCN**, and **PNDI‐SVSCN**.

**Scheme** **1** shows molecular structures and the synthetic routes of **PNDI‐BTCN, PNDI‐TVTCN**, and **PNDI‐SVSCN**. Compound SVS‐Br was synthesized according to methods reported by Cheng et al.^[^ ^31^ ^]^ Then SVSCN was obtained under the condition of Zn(CN)_2_ and Pd(PPh_3_)_4_. Compound SVSCN was treated with lithium diisopropylamide (LDA, 2 m) and quenched with trimethyltin chloride (1 m), affording monomer of SVSCN‐Sn successfully. The Stille polymerization was applied between monomer NDI‐C_10_C_12_ and BTCN‐Sn (TVTCN‐Sn or SVSCN‐Sn) to achieve copolymers of **PNDI‐BTCN**, **PNDI‐TVTCN**, and **PNDI‐SVSCN**. The molecular weights of polymers were evaluated with high‐temperature gel permeation chromatography (GPC) at 150 °C, calibrated with a polystyrene standard. The number‐average molecular weights (*M*_n_) and the polydispersity (PDI) of **PNDI‐BTCN**, **PNDI‐TVTCN**, and **PNDI‐SVSCN** are 13.67 kDa/1.92, 21.05 kDa/2.01, and 30.78 kDa/2.24, respectively. By performing thermogravimetric analysis and differential scanning calorimetry characterizations (Figure S1, Supporting Information), we also studied the thermal properties of all the copolymers, and the results indicate that the three copolymers show excellent thermal stability with the decomposition temperature up to 400 °C. In addition, **PNDI‐TVTCN** shows a couple of peaks at 273 and 303 °C, which correspond to the freezing and melting points, respectively. However, there are no apparent peaks for **PNDI‐BTCN** and **PNDI‐SVSCN**, indicating both the polymers do not proceed obvious melting and solidification process during this temperature range.

### 2.2 Optical Properties and Energy‐Level Evaluation

**Figure 2:**
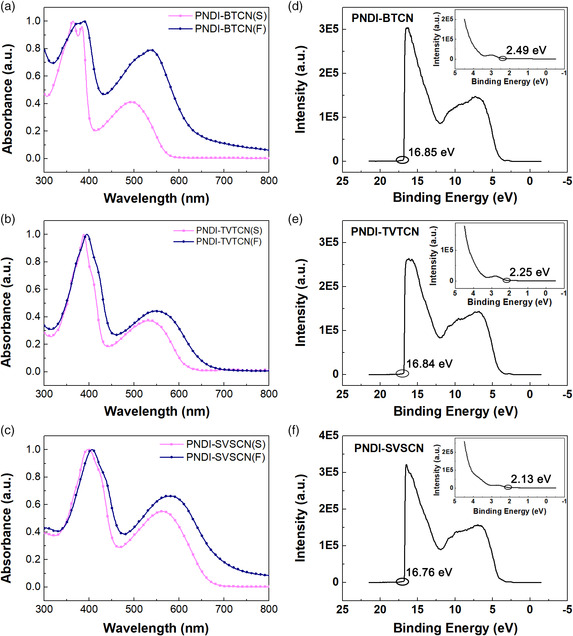
UV–vis absorption spectra and ultraviolet photoelectron spectroscopy of a,d) **PNDI‐BTCN**, b,e) **PNDI‐TVTCN**, and c,f) **PNDI‐SVSCN**.

We investigated the photophysical properties of this series of polymers by conducting UV–vis absorption characterization, and the results are shown in **Figure** **2**. Obviously, all copolymers display similar absorption profiles. **PNDI‐BTCN**, **PNDI‐TVTCN**, and **PNDI‐SVSCN** exhibit typical dual absorption bands. And this dual‐band absorption profiles are typical features of NDI‐based polymers. The high‐energy absorption band is attributed to *π*−π* transition, whereas the low‐energy absorption band corresponds to the interaction between the NDI section and the cyano‐substituted aromatic section. In addition, there is an obvious absorption bathochromic‐shift from the solution to the thin film for all the three polymers, especially for the low‐energy absorption band. Such shifts are ascribed to a more ordered packing and stronger interaction among molecules in the film state. Owing to the stronger electron‐donating ability of TVTCN and SVSCN than that of BTCN, the absorption peaks of **PNDI‐TVTCN** and **PNDI‐SVSCN** are bathochromic‐shift compared with **PNDI‐BTCN**. Estimated from the onsets of film absorption spectra, the optical gaps of **PNDI‐BTCN**, **PNDI‐TVTCN**, and **PNDI‐SVSCN** are 1.98, 1.89, and 1.78 eV, respectively.

According to the working principle of OFET, the carrier transport type is closely related to the frontier orbital energy levels of molecule. Therefore, we conducted ultraviolet photoelectron spectroscopy (UPS) characterization of polymer thin films on silica substrates and the results are shown in Figure 2. Apparently, the *E*_cutoff_ and *E*_H,onset_ of **PNDI‐BTCN**, **PNDI‐TVTCN**, and **PNDI‐SVSCN** are 16.85/2.49, 16.84/2.25, and 16.76/2.13 eV, respectively. By adopting the equation of IP = *hv* − (*E*_cutoff_ − *E*_H,onset_) eV, the ionization potential (IP) was determined to be 6.86 eV for **PNDI‐BTCN**, 6.63 eV for **PNDI‐TVTCN**, and 6.59 eV for **PNDI‐SVSCN**. Based on the optical energy gap obtained earlier, the LUMO energy levels of **PNDI‐BTCN**, **PNDI‐TVTCN**, and **PNDI‐SVSCN** are calculated to be −4.88, −4.74, and −4.81 eV, respectively. We also conducted cyclic voltammetry measurements to detect the frontier orbital energy levels with a three‐electrode system, in which a glass carbon electrode, a wire of platinum, and an Ag/AgCl electrode act as the working, the counter, and the reference electrodes, respectively. The cyclic voltammetry curve indicates that all the polymers exhibit obvious reduction peak, and there is no discernible oxidation peak (Figure S2, Supporting Information). The emergence of the reduction peak indicates the obtained copolymers undergo n‐doping facilely, and the absence of the oxidation peaks suggests that all the three polymers cannot go on p‐doping effectively. Based on the onset of the first reduction peak, the LUMO energy levels of **PNDI‐BTCN**, **PNDI‐TVTCN**, and **PNDI‐SVSCN** are estimated to be −4.09, −4.16, and −4.15 eV, respectively. Compared with the bithiophene derivative of P(NDI2OD‐T2), **PNDI‐BTCN** exhibits lower LUMO energy level by 0.09 eV, attributing to the introduction of the strong electron‐withdrawing cyano group. Moreover, the further decreased LUMO energy levels of **PNDI‐TVTCN** and **PNDI‐SVSCN** are possibly attributed to the enhanced intramolecular interaction due to the insertion of double bond section. The LUMO energy levels calculated based on IPs and optical gaps are deeper than those estimated from the reduction peaks and the large difference between the two methods is ascribed to the hole–electron binding energy. The frontier orbital energy levels imply that all the polymers would perform as n‐type semiconductors (**Table** **1**).

**Table 1:** Summary of molecular weights, photophysical properties, ionization potential, and cyclic voltammetry characterizations for **PNDI‐BTCN**, **PNDI‐TVTCN**, and **PNDI‐SVSCN**

Polymer	*M*_n_ [KDa]a)	PDIa)	$E_{\text{film}}^{\text{onset}}$ [eV]	$E_{\text{film}}^{\text{gap}}$ [eV]b)	IP [eV]c)	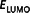 [eV]d)	$E_{\text{red}}^{\text{onset}}$ [V]	$E_{\text{LUMO}}^{\text{CV}}$ [eV]e)
PNDI‐BTCN	13.67	1.92	626	1.98	6.86	−4.88	−0.31	−4.09
PNDI‐TVTCN	21.05	2.01	657	1.89	6.63	−4.74	−0.24	−4.16
PNDI‐SVSCN	30.78	2.24	695	1.78	6.59	−4.81	−0.25	−4.15

a) Determined by GPC at 150 °C;

b) $E_{\text{film}}^{\text{gap}}$ = 1240/$E_{\text{film}}^{\text{onset}}$;

c) IP = *hv *− (*E*_cutoff_ − *E*_H,onset_) eV;

d) *E*_LUMO_ = − (IP − $E_{\text{film}}^{\text{gap}}$) eV;

e) $E_{\text{LUMO}}^{\text{CV}}$ = − ($E_{\text{red}}^{\text{onset}}$ + 4.40) eV.

### 2.3 Theoretical Simulation

**Figure 3:**
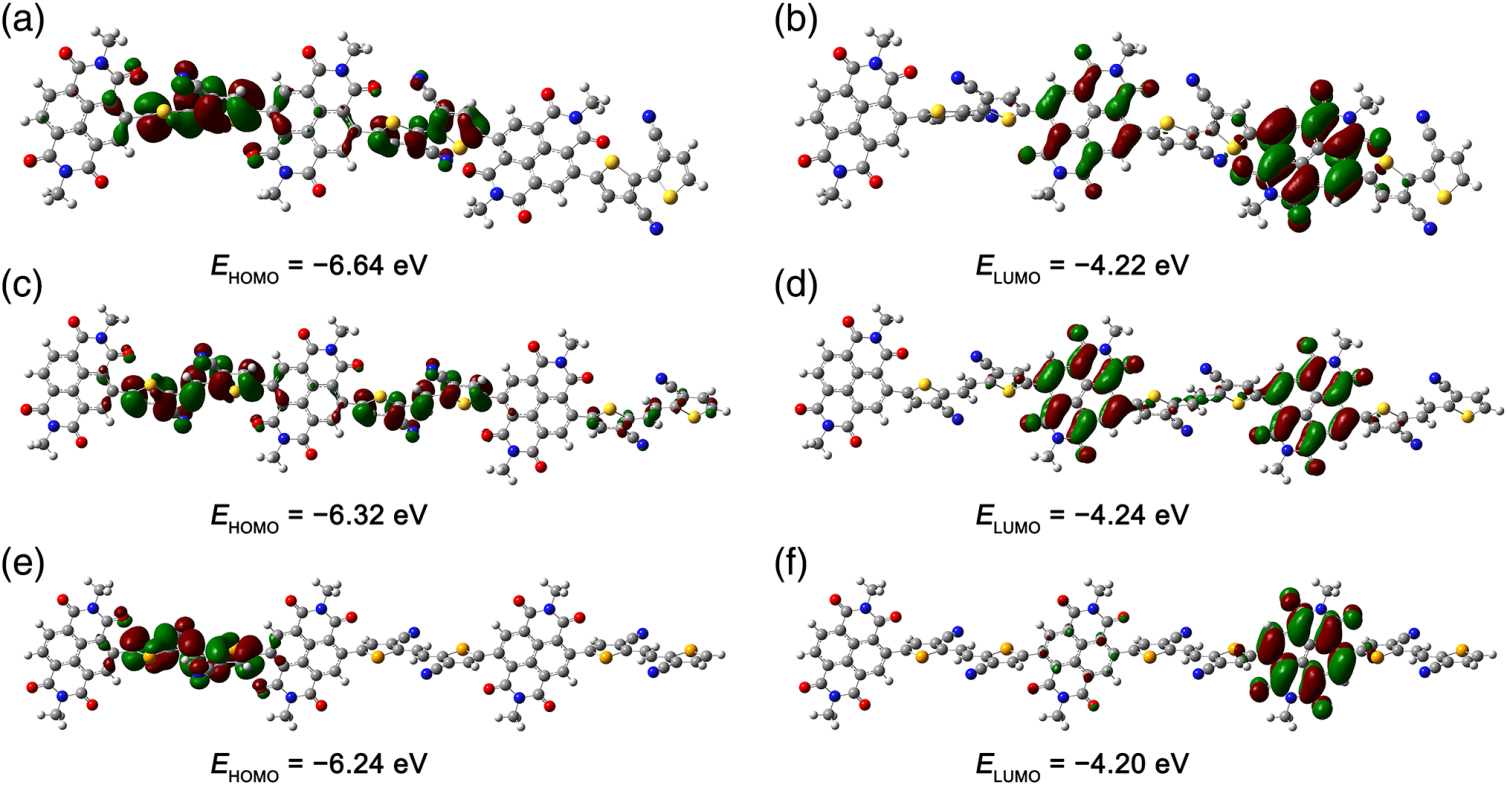
Frontier molecular orbitals and the calculated energy levels of model trimers of **PNDI‐BTCN** (up), **PNDI‐TVTCN** (middle), and **PNDI‐SVSCN** (down).

Molecular simulation on model trimers of **PNDI‐BTCN**, **PNDI‐TVTCN**, and **PNDI‐SVSCN** was conducted to investigate molecular geometries and the electronic structures with a hybrid B3LYP correlation functional and 6–31 + G (*d*) basis set. The alkyl chains were replaced by methyl groups for simplicity. Figure S3, Supporting Information, shows the simulated molecular geometries and **Figure** **3** displays the frontier orbitals of model trimers. Apparently, the molecule structures of all the synthesized copolymers are distorted, resulting from the large dihedral angle between NDI units and the cyano‐substituted aromatic blocks (60.36° for **PNDI‐BTCN**, 56.09° for **PNDI‐TVTCN**, and 86.93° for **PNDI‐SVSCN**). In addition, the nonplanar BTCN unit causes the molecular skeleton of **PNDI‐BTCN** to be more distorted than those of **PNDI‐TVTCN** and **PNDI‐SVSCN**. The twisted molecular backbone contributes to the localization of frontier molecular orbitals as revealed by Figure 3. In other words, the NDI section dominates the LUMO energy level and the cyano‐substituted aromatic blocks determine the highest occupied molecular orbital (HOMO) energy level. The HOMO/LUMO energy levels of model trimers of **PNDI‐BTCN**, **PNDI‐TVTCN**, and **PNDI‐SVSCN** are −6.64/−4.22, −6.32/−4.24, and −6.24/−4.20 eV, respectively, consistent with the experimental results. According to the experimental and calculated energy levels, we speculate that all the copolymers will behave as unipolar n‐type semiconductors because such energy levels block hole injection and facilitate electron injection at the same time.

### 2.4 Charge Transport Characteristics

**Figure 4:**
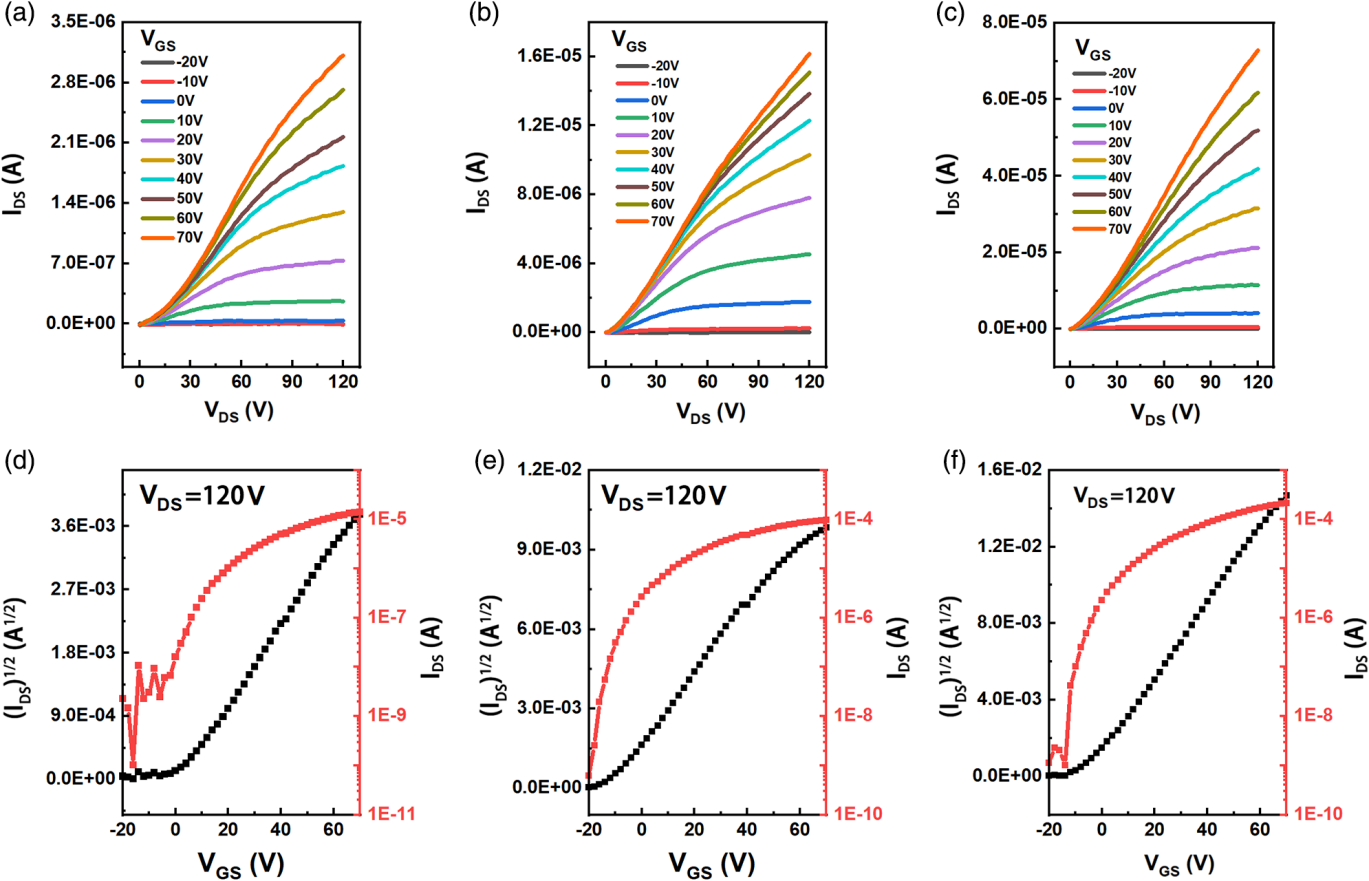
Typical output and transfer curves of OFETs based on a,d) **PNDI‐BTCN**, b,e) **PNDI‐TVTCN**, and c,f) **PNDI‐SVSCN**.

To investigate the semiconducting performance, we fabricated OFETs with a top‐gate/bottom‐contact (TG/BC) configuration based on the synthesized polymeric materials. The detailed fabrication and measurement process are provided in the Experimental Section. **Figure** **4** shows the typical output and transfer curves of the fabricated OFET devices. The OFETs based on **PNDI‐BTCN**, **PNDI‐TVTCN**, and **PNDI‐SVSCN** show representative n‐type carrier transport characteristics and the corresponding semiconducting performances are shown in **Table** **2**. The exclusive n‐type semiconducting properties are attributed to the low frontier orbital energy levels, merely facilitating the electron injection, which is consistent with the results of ultraviolet photoelectron spectroscopy and cyclic voltammetry characterizations. For OFETs, thermal annealing treatment is beneficial for molecular rearrangement into a crystalline state, and further elevating the carrier mobility. The optimal annealing temperature was found to be 200 °C (Figure S4, Supporting Information), and the corresponding highest mobility are 0.12, 0.16, and 0.20 cm^2^ V^−1^ s^−1^ for **PNDI‐BTCN**, **PNDI‐TVTCN**, and **PNDI‐SVSCN**, respectively.

**Table 2:** Summary of device performances for the OFETs based on **PNDI‐BTCN**, **PNDI‐TVTCN**, and **PNDI‐SVSCN** annealed at 200 °C

Polymer	*μ*_max_ [cm^2^ V^−1^ s^−1^]	*μ*_ave_ [cm^2^ V^−1^ s^−1^]	*I*_on/off_	*V*_th_ [V]
PNDI‐BTCN	0.12	0.08	10^5^	−9
PNDI‐TVTCN	0.16	0.12	10^5^	−12
PNDI‐SVSCN	0.20	0.15	10^5^	−10

### 2.5 Film Morphology and Microstructural Analysis

**Figure 5:**
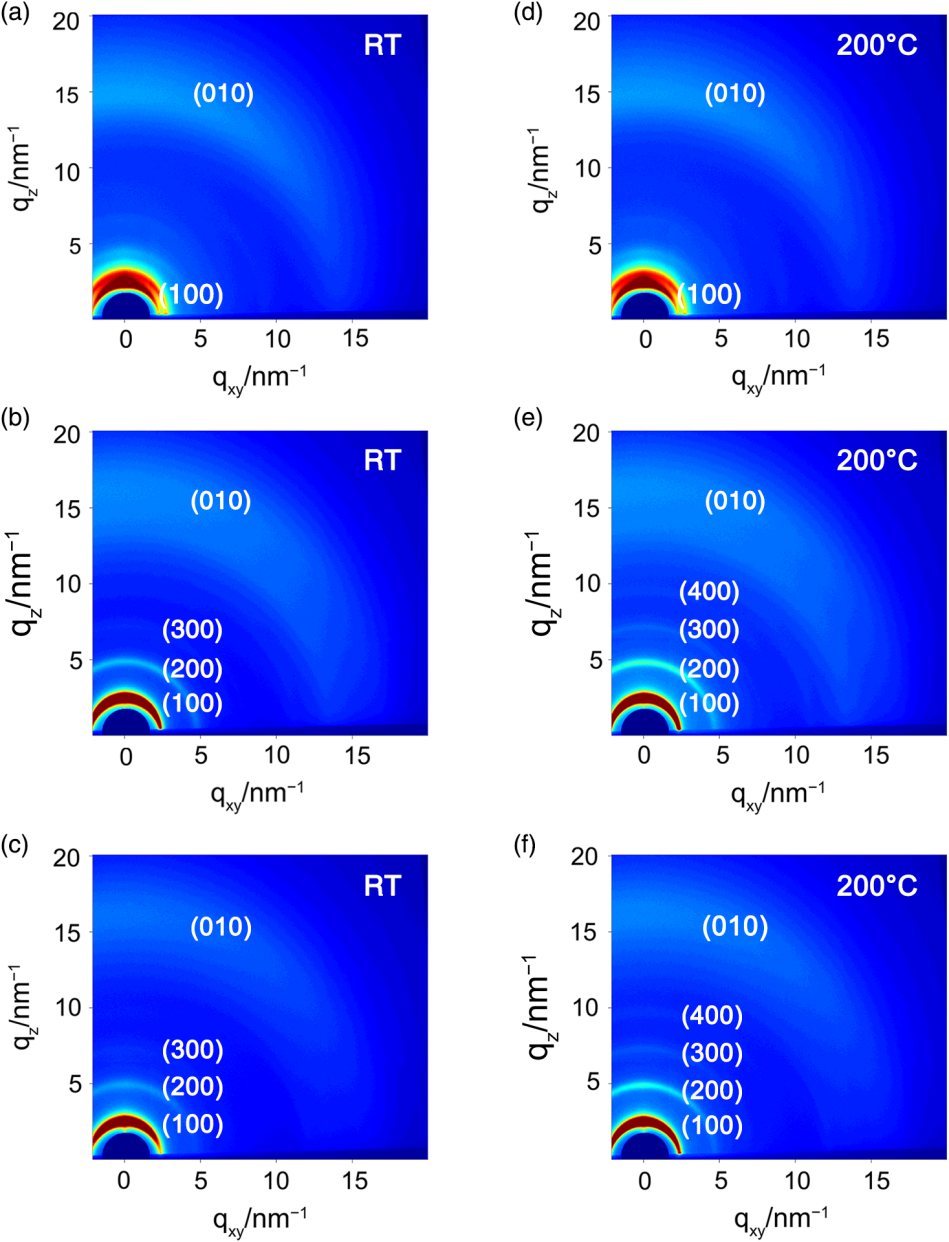
GIXRD patterns of thin films of a,d) **PNDI‐BTCN**, b,e) **PNDI‐TVTCN**, and c,f) **PNDI‐SVSCN** before and after thermal annealing treatment.

The molecular order and crystallinity of polymer thin‐films play a significant role in the semiconducting performance.^[^ ^32^, ^33^ ^]^ Therefore, we performed 2D grazing incidence X‐ray diffraction (2D‐GIXRD) of the polymer thin films to study the crystallinity and the molecular orientation relative to substrate. As shown in **Figure** **5**, the pristine films of **PNDI‐BTCN** show weak diffraction peak in the out‐of‐plane direction, while **PNDI‐TVTCN** and **PNDI‐SVSCN** thin film exhibit obvious (*h*00) diffraction peaks up to three orders. The arc‐shaped of (100) peak indicates that the crystallites are oriented randomly, possibly attributing to the twisted polymer skeleton. In addition, a weak (010) peak also appears in the out‐of‐plane direction (Figure 5 and Figure S5, Supporting Information), representing the face‐on orientation. After thermal annealing treatment at 200 °C, the diffraction intensity gets enhanced. Moreover, as for **PNDI‐TVTCN** and **PNDI‐SVSCN**, one more diffraction peak appears, demonstrating the enhanced crystallinity after annealing treatment, partially contributing to the slightly higher mobility than that of **PNDI‐BTCN**. Based on the (010) peaks, we calculated the *π*–*π* stacking distances. The *π–π* stacking distances for pristine and annealed films are 4.22/4.20 Å for **PNDI‐BTCN**, 3.80/3.75 Å for **PNDI‐TVTCN**, 3.83/3.80 Å for **PNDI‐SVSCN** (Figure S5, Supporting Information). Compared with **PNDI‐BTCN**, the denser arrangement of **PNDI‐TVTCN** and **PNDI‐SVSCN** could be another reason for their slightly higher semiconducting performance. In addition, we also performed pole figure analysis to determine the orientation distribution of the crystallites with respect to the substrate (Figure S6, Supporting Information). The results demonstrate that all the thin films adopt edge‐on arrangement predominately. After thermal annealing treatment, the orientation distributions have no obvious change, suggesting the thermal annealing process almost has no impact on the orientation distributions for this series materials.

**Figure 6:**
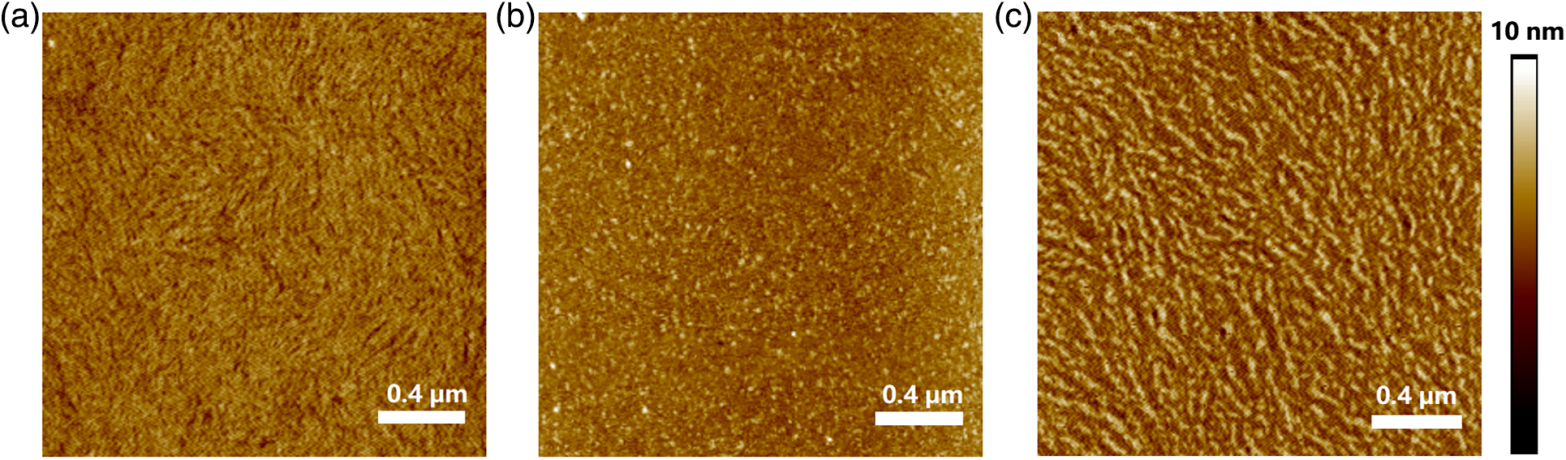
AFM images of polymer films of a) **PNDI‐BTCN**, b) **PNDI‐TVTCN**, and c) **PNDI‐SVSCN**.

We also explored the surface morphology of polymer thin films annealed at 200 °C by conducting atomic force microscope (AFM) characterizations. **Figure** **6** shows the corresponding AFM images. Apparently, the introduction of BTCN, TVTCN, and SVSCN contributes to different surface morphologies for the final copolymers. For **PNDI‐BTCN**, the annealed film has compact fibrous structure. In contrast, **PNDI‐TVTCN** film shows amorphous structure. Compared with **PNDI‐BTCN** and **PNDI‐TVTCN**, **PNDI‐SVSCN** film exhibits a more obvious fibrous structure, benefiting the carrier transporting. The aforementioned investigations confirm that both the crystallinity and surface morphology affect the final semiconducting performance. Thin film of **PNDI‐SVSCN** shows good crystallinity and surface morphology simultaneously, and contributing to the best semiconducting performance of the series of polymers.

## 3 Conclusion

In this article, three cyano‐substituted monomers, i.e., BTCN, TVTCN, and SVSCN, were designed and synthesized and further copolymerized with NDI monomer, providing **PNDI‐BTCN**, **PNDI‐TVTCN**, and **PNDI‐SVSCN**, respectively. Due to the synergetic effect of the strong electron‐deficient NDI unit and cyano‐substituted aromatic blocks, all the resulted copolymers possess low LUMO energy levels of blow −4.0 eV, and thus the synthesized polymeric materials exhibit unipolar n‐type semiconducting performance. The highest mobilities of devices based on **PNDI‐BTCN**, **PNDI‐TVTCN**, and **PNDI‐SVSCN** are 0.12, 0.16, and 0.20 cm^2^ V^−1^ s^−1^, respectively. The theoretical calculations confirm that the molecular backbone of this series of copolymers is twisted severely and such phenomenon is ascribed to the large dihedral angle between NDI unit and the cyano‐substituted aromatic rings, further contributing to the HOMO and LUMO localized in BTCN (TVTCN or SVSCN) and NDI section, respectively. The superior semiconducting performance of **PNDI‐SVSCN** is attributed to its higher crystallinity and better surface morphology compared with those of **PNDI‐BTCN** and **PNDI‐TVTCN**, which are demonstrated by the GIXRD and AFM characterizations. This work proves that cyano‐substitution is an efficient strategy in constructing unipolar n‐type semiconductors.

## 4 Experimental Section

4.1

4.1.1

All the starting materials and reagents are commercially available and used directly without further purification unless otherwise noted. Tetrahydrofuran (THF) was dried in accordance with standard processes. 4,9‐dibromo‐2,7‐bis(2‐decyltetradecyl)benzo[*lmn*][3,8]phenanthroline‐1,3,6,8(2*H*,7*H*)‐tetraone (NDI‐C_10_C_12_),^[^ ^34^ ^]^ 5,5′‐bis(trimethylstannyl)‐[2,2′‐bithiophene]‐3,3′‐dicarbonitrile (BTCN‐Sn),^[^ ^35^ ^]^ (*E*)‐2,2′‐(ethene‐1,2‐diyl)bis(5‐(trimethylstannyl)thiophene‐3‐carbonitrile) (TVTCN‐Sn),^[^ ^29^ ^]^ and (*E*)‐1,2‐bis(3‐bromoselenophen‐2‐yl)ethene (SVS‐Br)^[^ ^31^ ^]^ were synthesized according to the procedures previously reported. To elevate the molecular weights of polymers, all the trimethyltin reagents were recrystallized before polymerization.

##### (E)‐1,2‐bis(3‐Cyanoselenophene‐2‐yl)Ethene (SVSCN)

To a two‐neck flask protected with argon, (*E*)‐1,2‐bis(3‐bromoselenophene‐2‐yl)ethene (SVS‐Br, 1.00 g, 2.25 mmol), Zn(CN)_2_ (582.02 mg, 4.96 mmol), Pd(PPh_3_)_4_ (260 mg, 0.23 mmol), and 10 mL of DMF were added successively. Then the mixture was heated to 110 °C and stirred overnight. After cooling the system to room temperature, the mixture was extracted with dichloromethane for three times, and the organic phase was washed with saturated brine and dried with anhydrous Na_2_SO_4_. Filtration was conducted and the solvent was removed on a rotary evaporator. Then column chromatography and recrystallization were further performed and the target compound was achieved as brown needle crystals (469 mg, 62%). ^1^H NMR (300 MHz, CDCl_3_, *δ*, ppm): 8.00 (d, 2H, *J* = 5.70 Hz), 7.42 (d, 2H, *J* = 5.70 Hz), 7.36 (s, 2H). ^13^C NMR (75MHz, CDCl_3_, *δ*, ppm): 157.17, 131.41, 131.13, 126.02, 115.41, 112.58. HR‐MS (EI): calcd for C_12_H_6_N_2_Se_2_, 337.8861; found, 337.8858.

##### (E)‐2,2'‐(Ethene‐1,2‐Diyl)bis(5‐(Trimethylstannyl)Selenophene‐3‐Carbonitrile) (SVSCN‐Sn)

To a two‐neck flask charged with argon, lithium diisopropylamine (LDA, 3.28 mmol, 1.64 mL) was added. The system was cooled to −78 °C with a liquid nitrogen–acetone bath, and then a THF solution of SVSCN (0.50 g, 1.49 mmol) was added dropwise. The mixture was stirred for 2 h at the same temperature, after which trimethyltin chloride (4.47 mmol, 4.47 mL) was added. Removing the liquid nitrogen–acetone bath, the system was stirred at the room temperature for another 2 h. The mixture was extracted with dichloromethane for three times and the organic phase was washed with saturated brine and dried with anhydrous Na_2_SO_4_. Filtration was conducted and the solvent was removed under reduced pressure. Column chromatography was conducted to purification, recrystallization was further performed, and the title compound was obtained as flake crystal (592 mg, 60%). ^1^H NMR (300 MHz, CD_2_Cl_2_, δ, ppm): 7.52 (t, 2H, *J* = 14.10 Hz), 7.35 (s, 2H), 0.41 (t, 18H, *J* = 28.50 Hz). ^13^C NMR (75 MHz, CD_2_Cl_2_, *δ*, ppm): 161.82, 149.31, 138.57, 125.95, 115.74, 113.43, −8.08. HR‐MALDI: calcd for C_18_H_22_N_2_Se_2_Sn_2_, 665.8157; found, 665.8171.

##### General Procedure for Polymerization and Purification

Monomer NDI‐C_10_C_12_ (0.10 mmol), BTCN‐Sn (TVTCN‐Sn or SVSCN‐Sn, 0.10 mmol), Pd_2_(dba)_3_ (dba = dibenzylideneacetone, 4.50 mg), and tri(*o*‐tolyl)phosphine (12.30 mg) were added to a Schlenk tube charged with argon successively, and then 5 mL of *o*‐dichlorobenzene was syringed into the tube. After which the system went through freeze–pump–thaw cycles at liquid nitrogen bath for three times to remove the oxygen thoroughly. The reaction solution was stirred for 72 h at 120 °C. The reaction was stopped and cooled to room temperature; the crude product was poured into 200 mL of methanol containing 2 mL HCl (aq. 6 m) and stirred for another 3 h; the solid was collected by filtration. Extraction was further conducted with methanol, acetone, and hexane to remove oligomers and the residual catalysts. The final product was obtained by using chloroform as the final extraction solvent and the molecular structures were determined by high‐temperature ^1^H NMR and elemental analysis.

##### PNDI‐BTCN

(131.14 mg, 80%). ^1^H NMR (500 MHz, d_2_‐C_2_D_2_Cl_4_, 373 K): *δ* (ppm) 8.87 (s, 2H), 7.60 (s, 2H), 4.19 (br, 4H), 2.09 (br, 2H), 1.49−1.31 (m, 80H), 0.93 (t, 12H). GPC: Mn = 13.67 kDa, PDI = 1.92. Anal. Calcd for (C_72_H_102_N_4_O_4_S_2_)n: C, 75.08; H, 8.93; N, 4.86. Found: C, 74.45; H, 8.90; N, 4.67.

##### PNDI‐TVTCN

(136.55 mg, 82%). ^1^H NMR (500 MHz, d_2_‐C_2_D_2_Cl_4_, 373 K): *δ* (ppm) 8.82 (s, 2H), 7.62 (s, 2H), 7.47 (s, 2H), 4.18 (br, 4H), 2.06 (br, 2H), 1.49−1.31 (m, 80H), 0.93 (t, 12H). GPC: Mn = 21.05 kDa, PDI = 2.01. Anal. Calcd for (C_74_H_104_N_4_O_4_S_2_)n: C, 75.46; H, 8.90; N, 4.76. Found: C, 75.00; H, 8.87; N, 4.58.

##### PNDI‐SVSCN

(137.21 mg, 78%). ^1^H NMR (500 MHz, d_2_‐C_2_D_2_Cl_4_, 373 K): *δ* (ppm) 8.85 (s, 2H),7.62 (d, 4H), 4.19 (br, 4H), 2.07 (br, 2H), 1.49−1.32 (m, 80H), 0.93 (H, 12H). GPC: Mn = 30.78 kDa, PDI = 2.24. Anal. Calcd for (C_74_H_104_N_4_O_4_Se_2_)n: C, 69.90; H, 8.24; N, 4.41. Found: C, 69.39; H, 8.26; N, 4.27.

##### Device Fabrication and Characterization

FETs were fabricated with a TG/BC configuration to investigate the semiconducting properties of all the synthesized copolymers. Highly doped n^++^‐Si/SiO_2_ substrates (300 nm) were used and the source and drain electrode (Au) were prepared by the photolithography technique. Then the substrates were soaked in acetone for 3 h and further treated with UV‐ozone for 20 min. Afterward, the treated substrates were subject to Piranha solution (H_2_SO_4_:H_2_O_2_ = 3:1) for 5 min to obtain hydroxylated surface and then modified with octadecyltrichlorosilane (OTS) in vacuum at 120 °C to form a self‐assembled layer. The modified substrates were washed with hexane, ethanol, and chloroform, and a solution of copolymer in *o*‐dichlorobenzene (8 mg mL^−1^) was deposited on the substrates by spin‐coating method, forming the semiconducting layer. Thermal annealing treatment at various temperatures was proceeded to optimize the semiconducting performance. Polymethyl methacrylate (PMMA) solution in *n*‐butyl acetate (60 mg mL^−1^) was spin‐coated on the top of the semiconducting layer, which was annealed at 85 °C to remove the residual solvent and act as the dielectric layer. The device was completed by evaporation of a thin layer of aluminum (Al) as the gate electrode (70 nm). The FET performances of all the fabricated devices were characterized directly in air with a Keithley 4200 SCS semiconductor parameter analyzer. All the mobilities were calculated in the saturated regime according to the following equation

(1) $$I_{\text{DS}}=(W/2L)C_{i}\mu {\left(V_{\text{GS}}-V_{\text{TH}}\right)}^{2}$$

where *L* and *W* correspond to the channel length and channel width, respectively. *C*_i_ is the insulator capacitance of PMMA per unit area. *V*_GS_ and *V*_TH_ are the gate voltage and the threshold voltage, respectively.

## Conflict of Interest

The authors declare no conflict of interest.

## Data Availability Statement

Research data are not shared.

## Supporting information

Supplementary Material
