# Supplementary material for: Incorporation of Cyano‐Substituted Aromatic Blocks into Naphthalene Diimide‐Based Copolymers: Toward Unipolar n‐Channel Field‐Effect Transistors
Source: Small Sci. 2021 Jul 16;1(9):2100016. doi: 10.1002/smsc.202100016 (PMC11936061; doi:10.1002/smsc.202100016)
Supplement: Supplementary file 1 — Supplementary Material [file SMSC-1-2100016-s001.pdf]

Incorporation of Cyano-Substituted Aromatic Blocks into Naphthalene Diimide-Based Copolymers: Towards Unipolar *n*-Channel Field-Effect Transistors

Congyuan Wei, Pan Xu, Weifeng Zhang, Yankai Zhou, Xuyang Wei, Yuanhui Zheng, Liping Wang, and Gui Yu**

General Measurements and Characterizations.

^{1}H NMR and ^{13}C NMR spectra were recorded on a Bruker AVANCE 300 instrument in deuterated chloroform ($CDCl_3$) or deuterated dichloromethane (CD_2Cl_2) at room temperature (RT). The high-temperature 1H NMR spectra were acquired on a Bruker AVANCE 500 instrument in deuterated 1,1,2,2-tetrachloroethane (d_2-$C_2D_2Cl_4$) at 373 K. High-resolution mass spectra (HR-EI) were recorded on a Shimadzu QP 2010 gas chromatograph mass spectrometer, and high-resolution matrix-assisted laser desorption/ ionization time-of-flight mass (HR-MALDI-TOF) spectra were performed on a 9.4T Solarix FT-ICR mass spectrometer. Elemental analyses were carried out by using a CARLO ERBA 1106 Elemental Analyzer. The number-average molecular weight (Mn), weight-average molecular weight (Mw), and polydispersity index (PDI) of copolymers were analyzed by using an Agilent Technologies PL-GPC220 series gel permeation chromatography (GPC) with 1,2,4-trichlorobenzene (TCB) as the eluent at 150 °C and calibrated against narrow polydispersity polystyrene standards. UV−vis absorption spectra were measured on a Jasco V-570 spectrophotometer. Cyclic voltammetric (CV) measurements were performed on a CHI660C electrochemistry

workstation with a scan rate of 50 mV s^{-1}. Thermogravimetric analyses (TGA) were conducted on a DTG-60 instrument under nitrogen flow, heating from RT to 550 °C with a heating rate of 10 °C min^{-1}. The surface morphologies of the thin films were investigated by using an atomic force microscope (AFM) operated in tapping mode. Two-dimensional grazing incident X-ray diffraction (2D-GIXRD) was applied to determine the molecular orientation and crystallinity. The GIXRD measurements were performed at BL14B1 beamline of Shanghai Synchrotron Radiation Facility. The incident beam energy was of 10.00 keV, and the data were collected with an incident angle of 0.50° on a MAR225 image-plate area detector.

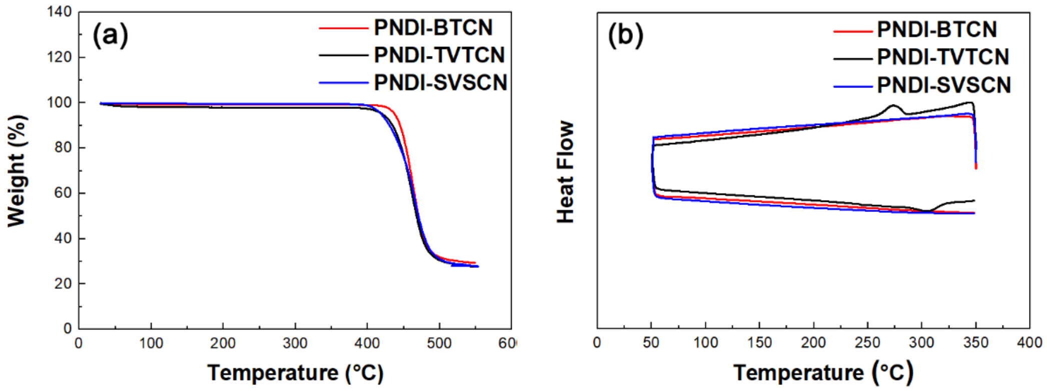

Figure S1. TGA (a) and DSC (b) curves of **PNDI-BTCN**, **PNDI-TVTCN**, and **PNDI-SVSCN**.

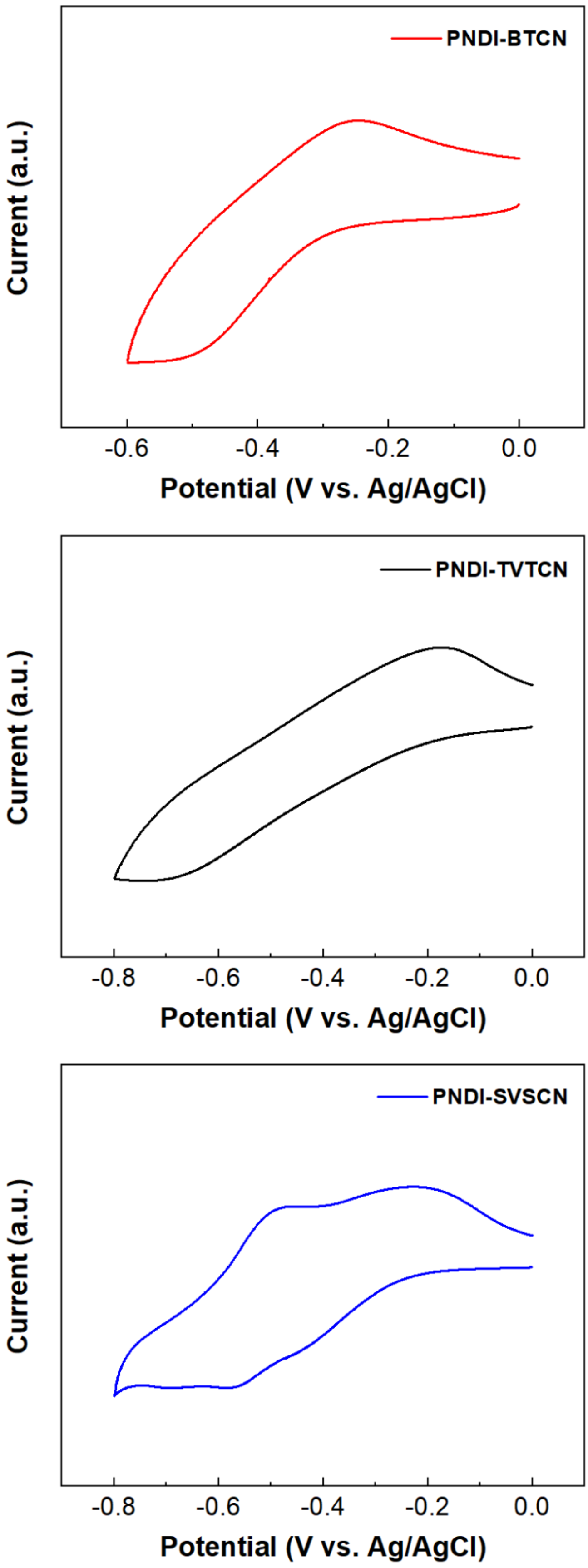

Figure S2. Cyclic voltammetry curves of **PNDI-BTCN**, **PNDI-TVTCN**, and **PNDI-SVSCN**.

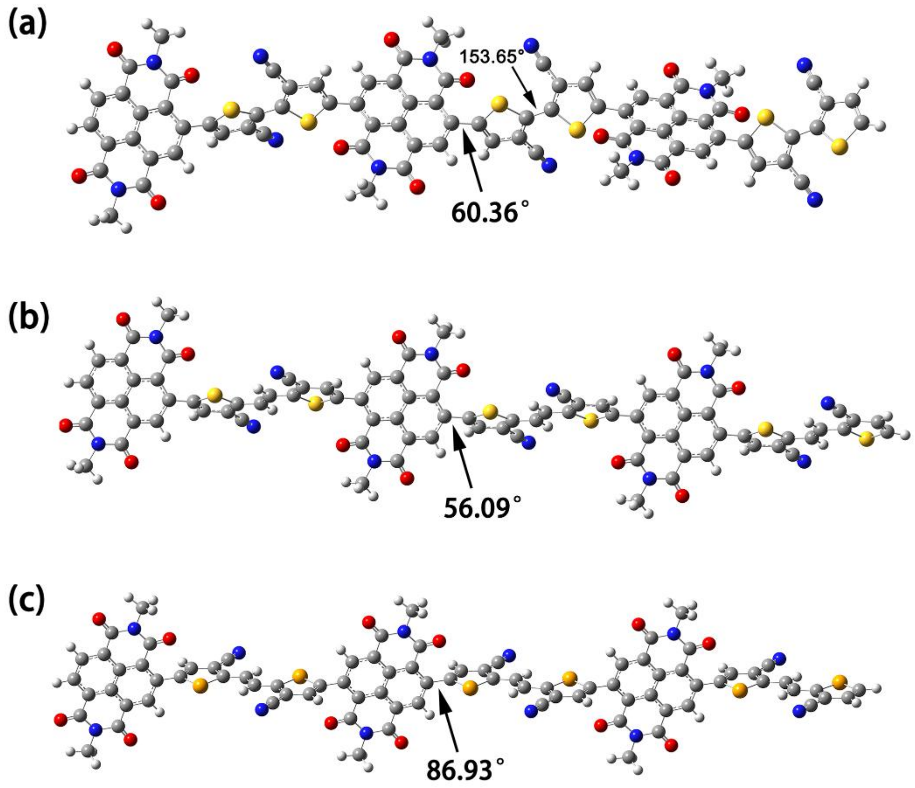

Figure S3. Molecular geometry of trimers of **PNDI-BTCN**, **PNDI-TVTCN**, and **PNDI-SVSCN**.

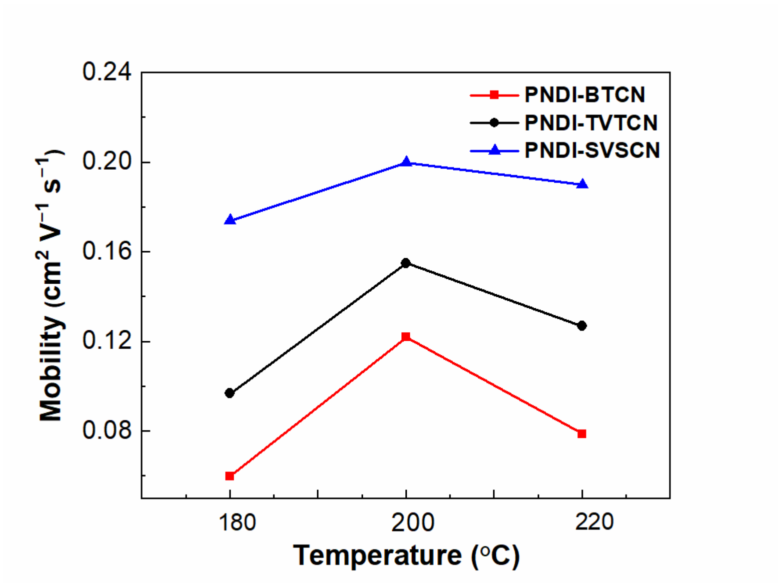

Figure S4. Temperature-dependent mobility of devices based on **PNDI-BTCN**, **PNDI-TVTCN**, and **PNDI-SVSCN**.

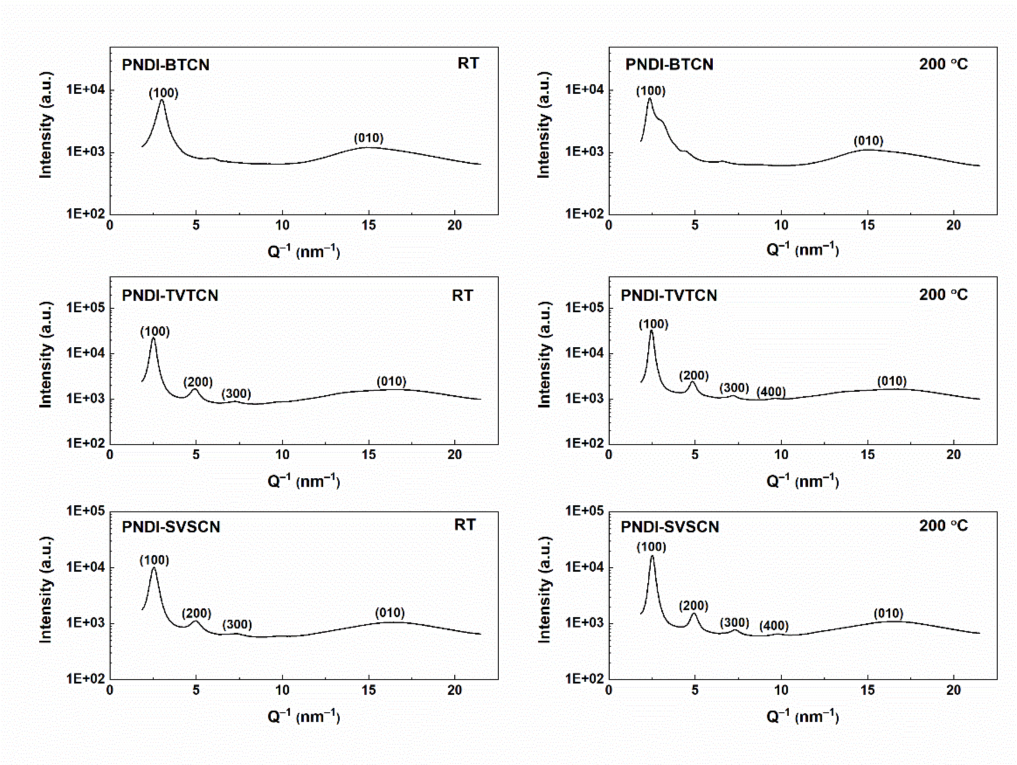

Figure S5. The 1D-XRD profiles in the out-of-plane extracted from 2D-GIXRD patterns of the **PNDI-BTCN**, **PNDI-TVTCN**, and **PNDI-SVSCN** thin films.

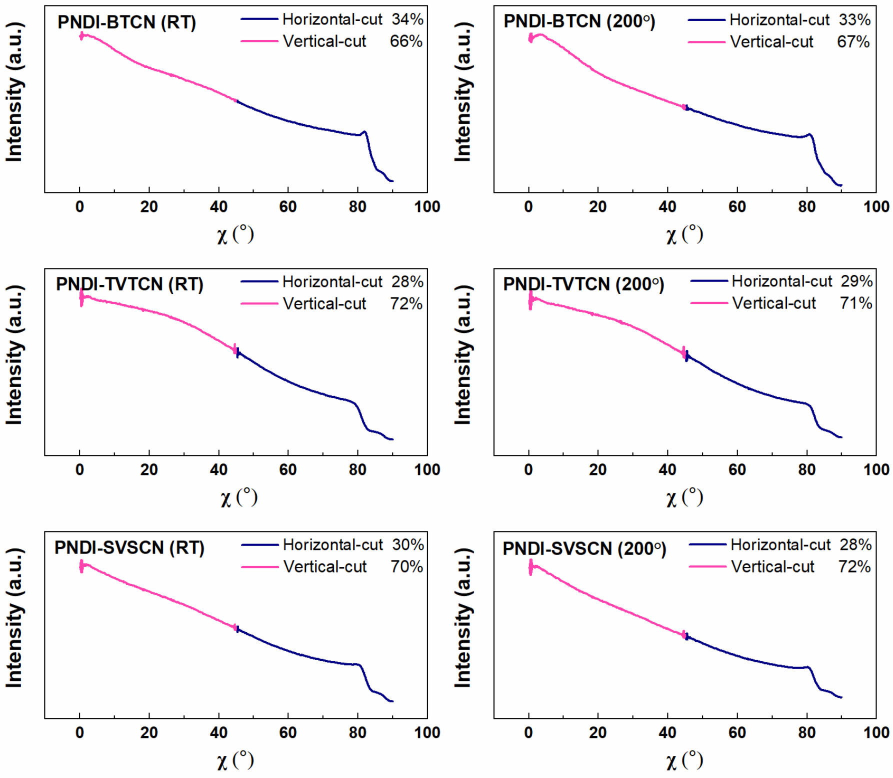

Figure S6. Pole figures of the (100) peaks in the range of 0-90° of the **PNDI-BTCN**, **PNDI-TVTCN**, and **PNDI-SVSCN** thin films before and after annealing treatments.

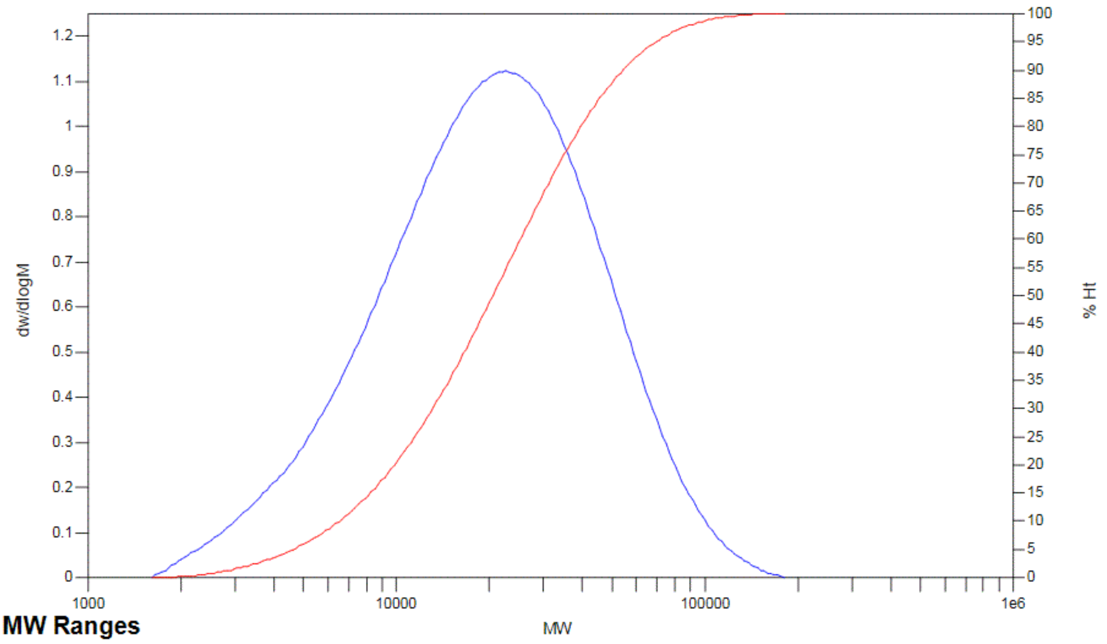

Figure S7. The size exclusion chromatographic (SEC) curve of **PNDI-BTCN.**

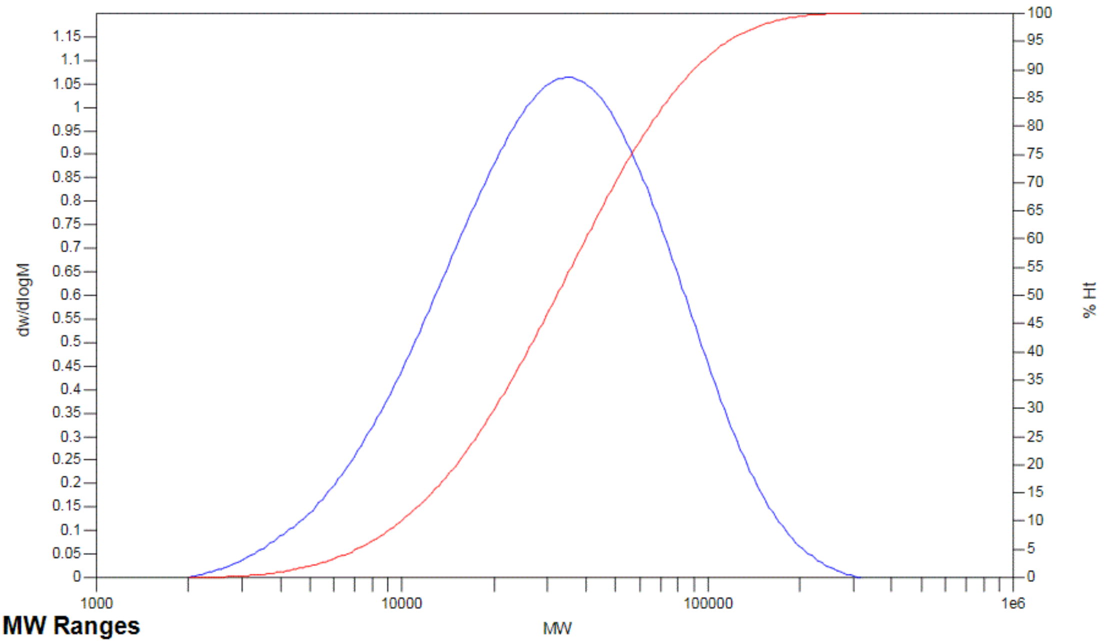

Figure S8. The size exclusion chromatographic (SEC) curve of **PNDI-TVTCN.**

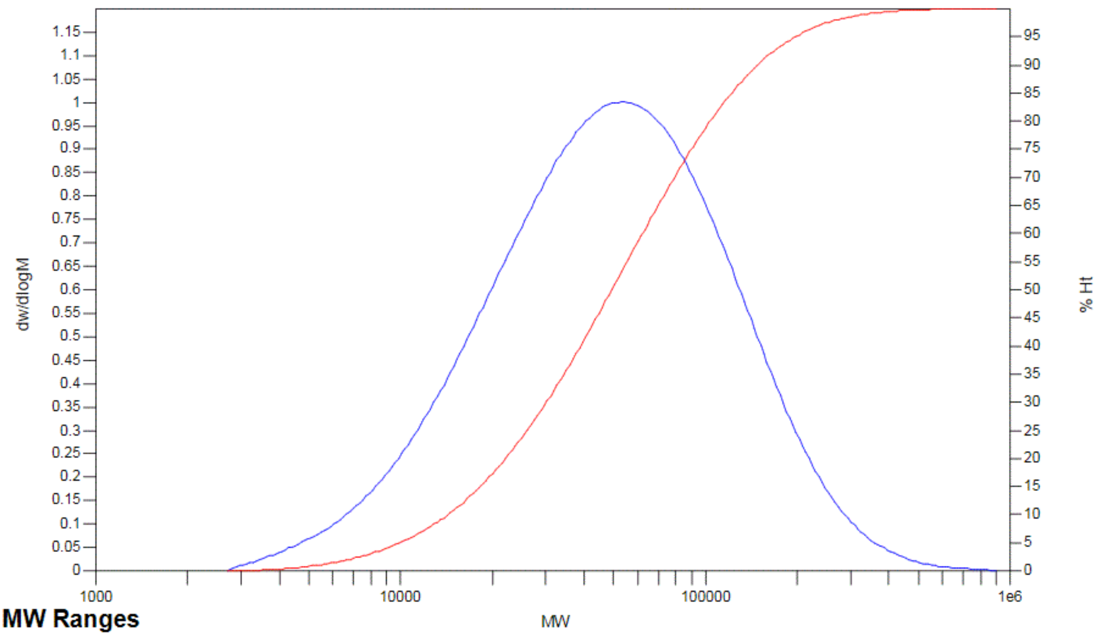

Figure S9. The size exclusion chromatographic (SEC) curve of **PNDI-SVSCN.**

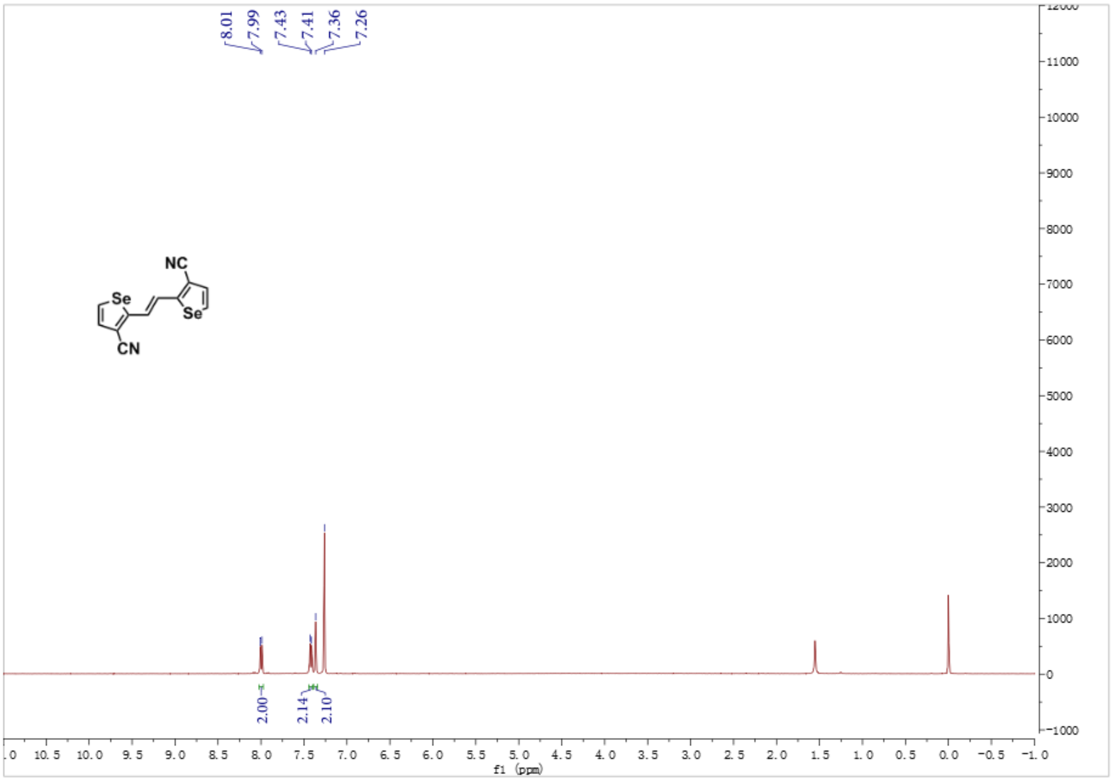

Figure S10. ^{1}H NMR spectrum of compound **SVSCN** (in $CDCl_3$)**.**

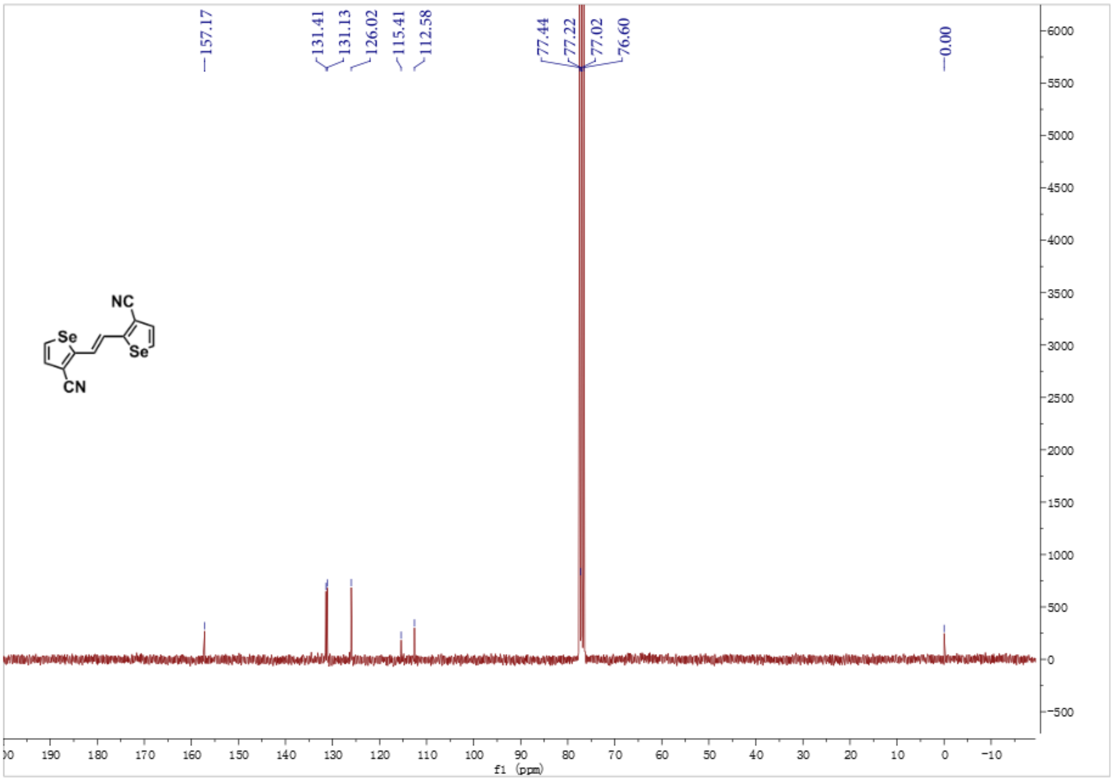

Figure S11. ^{13}C NMR spectrum of compound **SVSCN** (in $CDCl_3$).

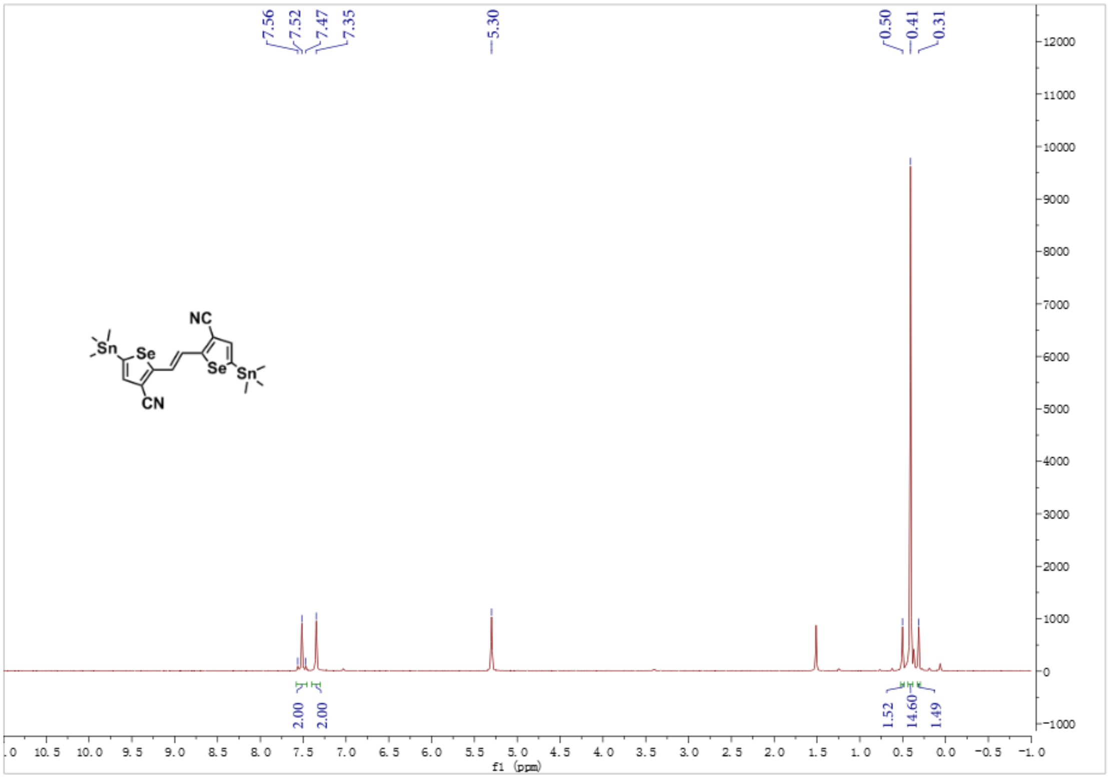

Figure S12. ^{1}H NMR spectrum of compound **SVSCN-Sn** (in CD_2Cl_2).

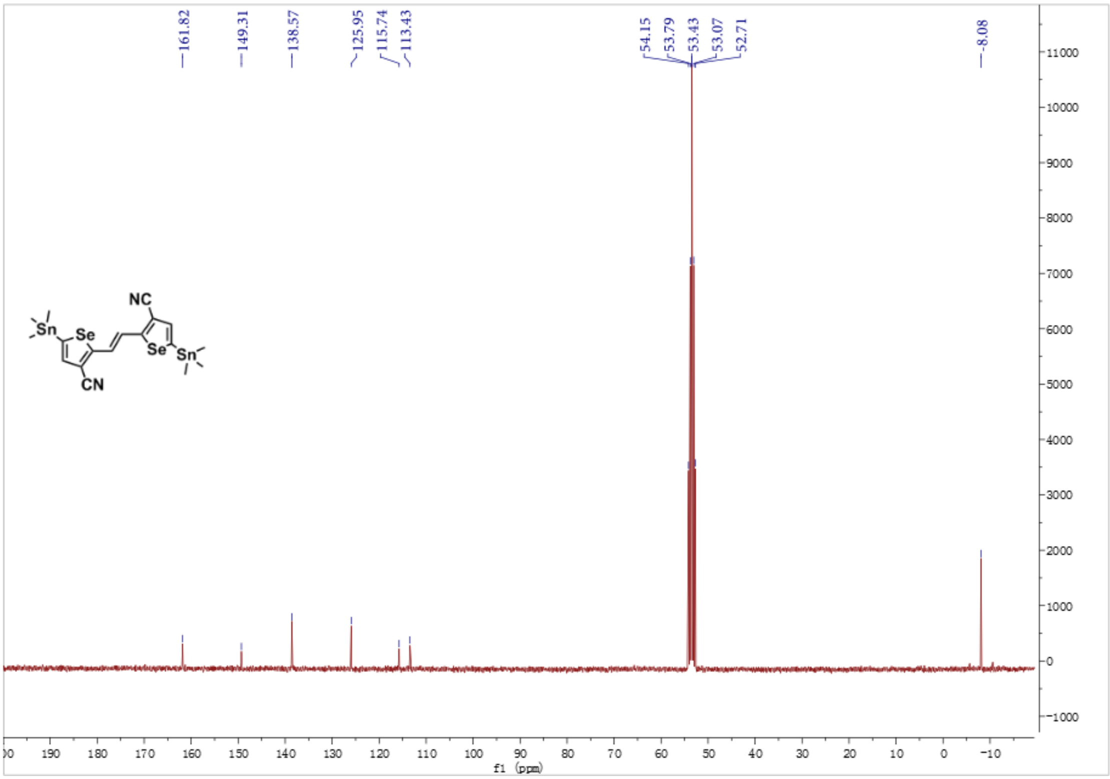

Figure S13. ^{13}C NMR spectrum of compound **SVSCN-Sn** (in CD_2Cl_2).

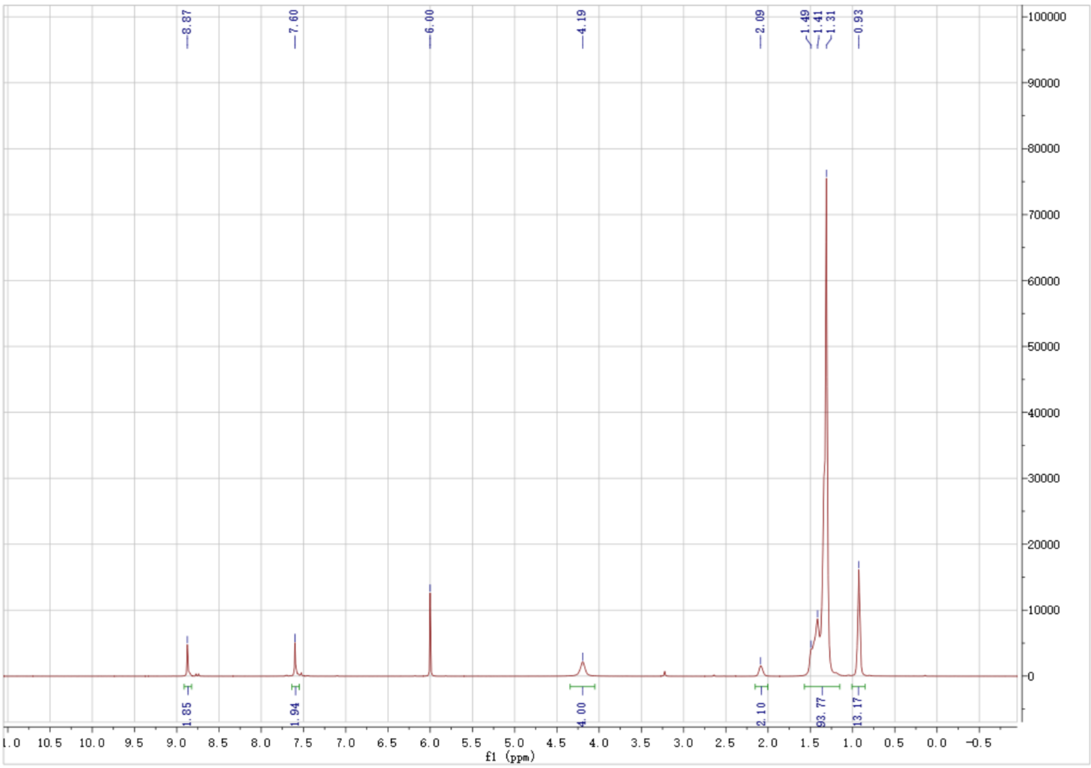

Figure S14. ^{1}H NMR spectrum of polymer **PNDI-BTCN** at 373 K (in d_2-$C_2D_2Cl_4$).

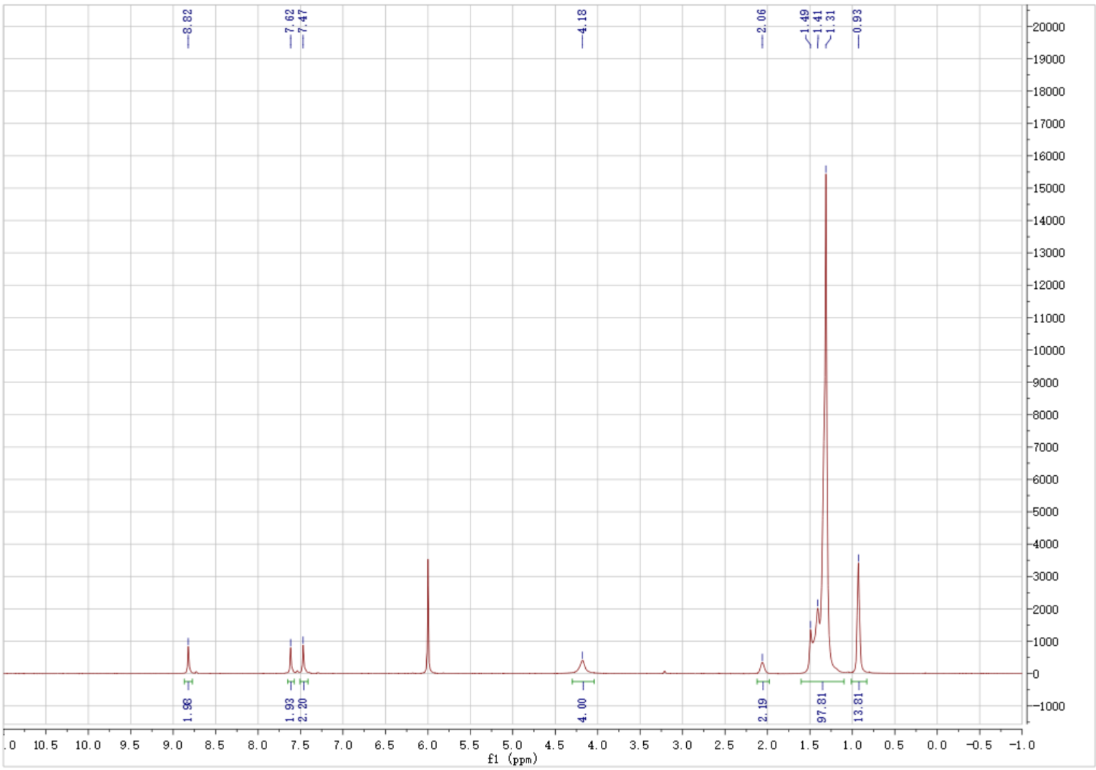

Figure S15. ^{1}H NMR spectrum of polymer **PNDI-TVTCN** at 373 K (in d_2-$C_2D_2Cl_4$).

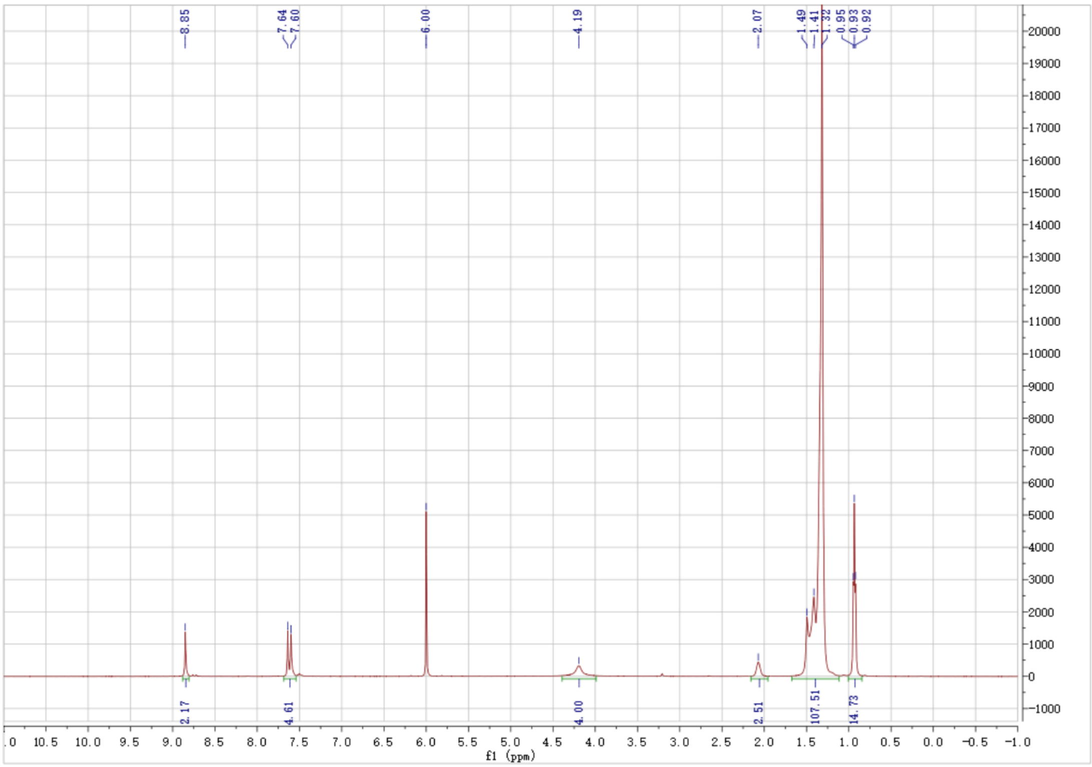

Figure S16. ^{1}H NMR spectrum of polymer **PNDI-SVSCN** at 373 K (in d_2-$C_2D_2Cl_4$).
